# *Isoetes dubsii* and *Isoetes santacruzensis*, two new species from lowland areas in South America

**DOI:** 10.3897/phytokeys.131.36983

**Published:** 2019-09-05

**Authors:** Jovani B. de S. Pereira, José Tasso F. Guimaraes, Maurício T. C. Watanabe

**Affiliations:** 1 Instituto Tecnológico Vale, Rua Boaventura da Silva, 955, 66055-090, Belém, Pará, Brazil Instituto Tecnológico Vale Belém Brazil

**Keywords:** Aquatic plants, Bolivia, Brazil, herbarium collections, lycophytes, *
Isoetes
*, Pantanal wetlands, spores.

## Abstract

*Isoetes dubsii***sp. nov.** and *I. santacruzensis***sp. nov.**, two new species from lowland areas in South America, are described, illustrated and compared to similar species. *Isoetes dubsii* can be distinguished from other species of the Brazilian Pantanal wetlands by a set of characters including leaves that are long, flexuous and trigonal in transverse section, tri-lobate stems, rudimentary velum, pustulate megaspores of 310‒390 µm diameter and laesurae of the megaspore at least four times wider than high. *Isoetes santacruzensis* has flexuous, filiform leaves, 0.4–0.8 mm wide at mid length and reaching up to 15 cm long, black or reddish-black sporangia, sclerified phyllopodia and sparsely verrucate megaspores of 320‒390 µm in diameter. We also include a key for species from the Brazilian Pantanal wetlands and Bolivia and spore images for all species that are discussed. *Isoetes dubsii* and *I. santacruzensis* are only known from their type localities and they may deserve special attention concerning their conservation status. However, based on our current knowledge on these species and according to IUCN Red List criteria, they are assessed here as data deficient (DD).

## Introduction

Herbarium collections are amongst the most important tools for obtaining information about the composition, distribution and content of plant diversity in a given region (Nualart et al. 2017). They represent a cumulative body of knowledge, which has been generated over time. It is also well known that many undescribed species reside in existing herbarium collections (Bebber et al. 2010).

*Isoetes* L. is the unique extant genus of heterosporous lycophytes in the Isoetales (PPG I 2016). *Isoetes* is morphologically well defined and readily distinguishable from any other group of vascular plant by its narrow leaves containing four air-chambers, a single sunken adaxial sporangium covered by a velum and sporangial trabeculae (Taylor et al. 2016). The genus comprises about 250 species (Troia et al. 2016). South America is one of its centres of taxonomic diversity (Troia et al. 2016) with an estimated 64 species (Hickey et al. 2003). Most of the species are narrowly endemic and they occur as aquatic or terrestrial plants in wet soils (Pfeiffer 1922). However, *Isoetes* species are notorious for the difficulties they present in identification, which are partially associated with morphological simplicity (Taylor and Hickey 1992).

Difficulties in the identification have frequently led many *Isoetes* specimens to be deposited in herbaria without determinations or with wrong determinations (Troia and Rouhan 2018). The sculpture of the megaspore is one of the most important characters in the taxonomy of the genus (Pfeiffer 1922) and, in many cases, scanning electron microscopy images (SEM) of megaspores are needed to identify species (Hickey 1986a). The use of SEM adds logistic difficulties in the taxonomy of the genus and, as a result, many *Isoetes* species remain unidentified and/or undescribed in herbarium collections.

Our recent efforts to access the diversity of *Isoetes* in South America has led us to consult herbarium collections where we discovered two species that we recognised as undescribed. One of the new species is from Bolivia and the other is from Pantanal wetlands in Brazil. We provide descriptions to distinguish these species, SEM images of mega- and microspores of species of these regions and a key for the identification of these new species.

## Material and methods

Fieldwork was carried out by Balthasar Dubs, a Swiss botanist and ornithologist who intensively collected plants in the Pantanal wetlands in Brazil and found *Isoetes dubsii* on 3 June 1988 in the Pantanal do Rio Negro (currently belonging to the municipalities of Aquidauana and Corumbá), in the state of Mato Grosso do Sul, mid-western Brazil. We also tried to locate this new species in the same area in November 2017. For *I. santacruzensis*, fieldwork was carried out by Timothy J. Killeen on 11 November 1994 in the province of Ñuflo de Chávez, Department of Santa Cruz, Bolivia.

Spore images were generated by transferring the spores to aluminium scanning electron microscope (SEM) stubs coated with a carbon adhesive. The stubs were then coated with gold-palladium-alloy in a sputter-coater for 180 sec, after which the spores were digitally imaged using a Zeiss SIGMA VP. The resulting images were adjusted in Photoshop for contrast and the background was altered to black. To measure the spores, we used a minimum of 20 spores per sporangium, from at least two sporangia. The spore measurements were taken using SEM. The terminology used for the description of the spores follows that of Punt et al. (2007), with some modification using Hickey (1986a).

## Taxonomic treatment

### Isoetes dubsii

Taxon classificationPlantaeIsoetalesIsoetaceae

J.B.S.Pereira sp. nov.

41EDF34DE65C57B880ADBB40A6A0E227

urn:lsid:ipni.org:names:77201652-1

Figs 1 , 2

#### Diagnosis.

*Isoetes dubsii* is distinguished from other species from the Brazilian Pantanal wetlands by a set of characters that include leaves that are long (90‒100 cm), flexuous and trigonal in the transverse section, tri-lobated stems, rudimentary velum, pustulate and small megaspores of 310‒390 µm in diameter (average 350 µm) and laesurae of the megaspore at least four times wider than high.

#### Type.

BRAZIL. Mato Grosso do Sul: Fazenda Salina, Pantanal do Rio Negro, 19°30'S, 56°10'W, 3 Jun 1988, *Dubs 829* (holotype: Z!).

**Figure 1.**
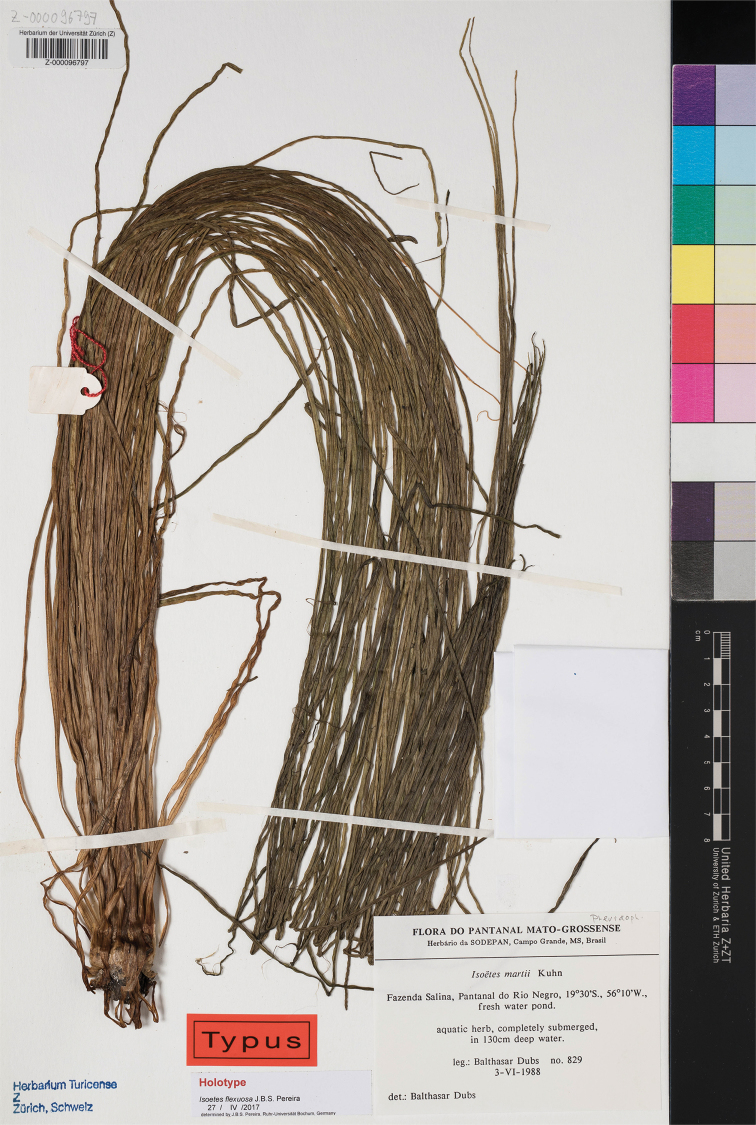
Type of *Isoetes dubsii* (image courtesy of the herbarium Z/ZT). Note that the name *Isoetes flexuosa* J.B.S. Pereira on the label sheet is a provisional, never published, name. Photographer: Franziska Schmid

**Figure 2.**
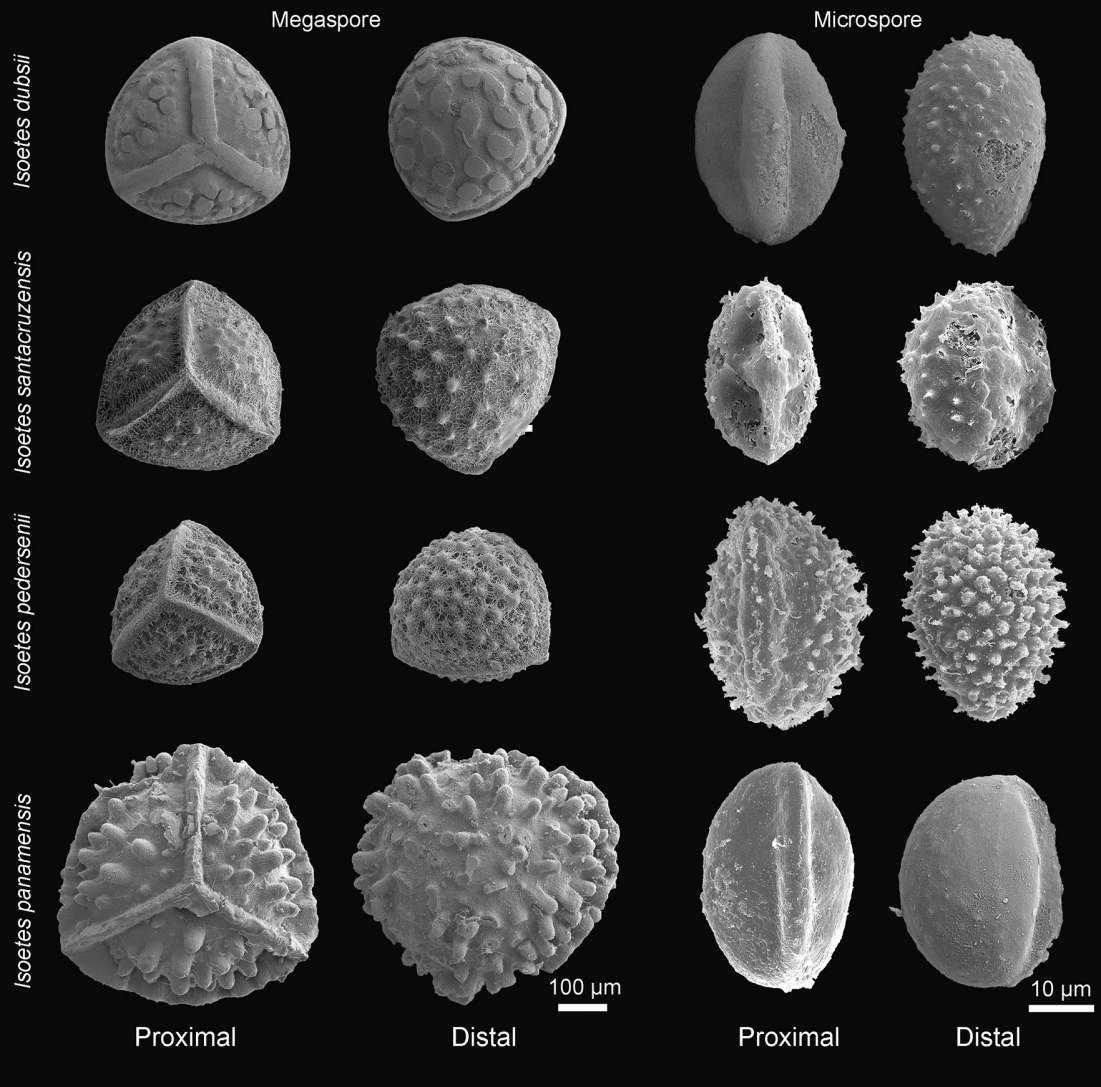
SEM images of the mega- and microspores of *Isoetes dubsii* (*Dubs 829*, Z/ZT), *I. santacruzensis* (*Killeen et al. 7131*, MO), *I. pedersenii* (*Troels 8105*, L) and *I. panamensis* (*Balansa 3294*, P).

#### Description.

Plants aquatic submerged, growing in fresh water ponds. Roots conspicuous, dichotomous. Stems globose, 3–lobate, ca. 2 cm wide. Leaves 90‒100 cm long, 0.2–0.3 cm wide at mid length, 50–60 per individual, linear, flexuous, erect, apex attenuate; alae 12–13 cm long, extending from the base ca. 1/10 of total leaf length, red-brown, membranaceous, apex attenuate; subula olive-green, trigonal. Labium present, persistent, 1.5–2.0 × 2.0–2.5 mm, cordate. Ligula not observed in herbarium material. Velum rudimentary. Sclerified phyllopodia absent. Sporangium at the base of the leaf, 8–12 × 3–4 mm, oblong, light brown, concolorous. Megaspores 310–390 µm in diameter (average = 350 µm, *n* = 20), trilete, white, not lustrous; proximal and distal surfaces pustulate, macrosculpture 3–8 × 22–45 µm, wider than high; laesurae 8–10 × 45–55 µm, at least four times wider than high. Microspores 31–36 µm long (average = 34 µm, *n* = 20), light brown, monolete, proximal surface smooth, distal surface sparsely echinate.

#### Distribution and habitat.

**Figure 3.**
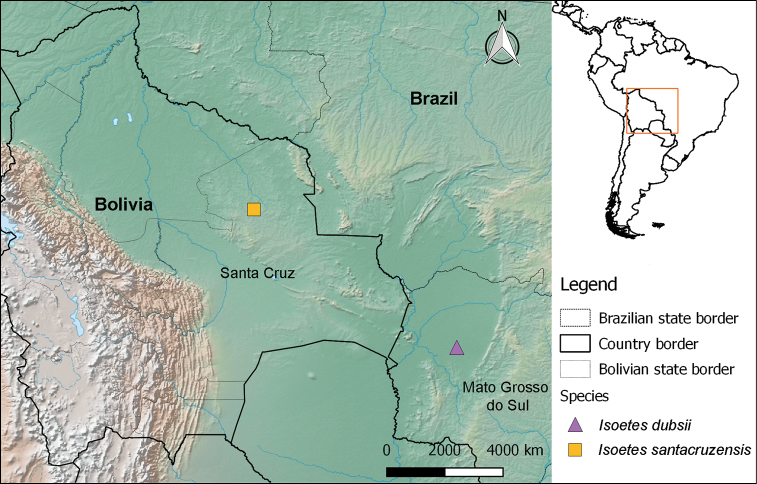
Map showing the type locations of *Isoetes dubsii* and *I. santacruzensis*.

This species is only known from its type locality (state of Mato Grosso do Sul, Brazil; Fig. 3), where it grows in a fresh water pond at ca. 100 m a.s.l. Although we tried to re-collect *Isoetes dubsii* in the area indicated by Balthasar Dubs, no additional collections have been made.

#### Etymology.

The specific epithet honours the Swiss ornithologist and botanist B. Dubs, for his valuable contributions to the knowledge of the flora and fauna of the Pantanal wetlands of Brazil. He was also the first naturalist to collect *Isoetes dubsii*.

#### Notes.

Amongst the species found in Brazilian Pantanal wetlands, *Isoetes panamensis* Maxon C.V. Morton *sensu lato* is similar to *I. dubsii* due to its long leaves up to 80 cm. However, the megaspores in *I. panamensis* are 380–600 µm (vs. 310–390 µm) in diameter and baculate (vs. pustulate) (Fig. 2).

#### Conservation status.

Since *I. dubsii* is currently known from a single locality, it may deserve special attention concerning its conservation status. However, based on our current knowledge on this species and according to IUCN Red List criteria (IUCN 2012), it is assessed here as data deficient (DD).

### Isoetes santacruzensis

Taxon classificationPlantaeIsoetalesIsoetaceae

J.B.S.Pereira sp. nov.

0CB9B8BCA03D58CAA7DB610008C588E0

urn:lsid:ipni.org:names:77201648-1

Figs 2 , 4

#### Diagnosis.

*Isoetes santacruzensis* is characterised by having flexuous, filiform leaves ranging from 0.4–0.8 mm wide at mid length and reaching up to 15 cm long, 15‒30 leaves per individual, black or reddish-black sporangia, sclerified phyllopodia present, sparsely verrucate megaspores of 320‒390 µm (average of 350 µm) in diameter.

#### Type.

BOLIVIA. Santa Cruz: Nuflo de Chaves, 15°32'40"S, 61°59'28"W, 450 m a.s.l., 11 Nov 1994, *Killeen et al. 7131* (holotype: MO!).

Plant aquatic partially submerged or ephemeral terrestrial in rocky granite outcrops. Roots conspicuous, dichotomous. Stems globose, 3–lobate, 0.8–1.2 cm wide. Leaves 6–15 cm long, 0.4–0.8 mm wide at mid length, 15–30 per individual, filiform, flexuous, laxly ascending, apex attenuate; alae 0.3–3.5 cm long, extending from the base ca. 1/5 of total leaf length, hyaline or light brown, membranaceous, apex attenuate; subula olive green, trigonal. Labium present, persistent, cordate, 0.2–0.5 × 0.6–0.9 mm. Ligule not observed in herbarium material. Velum rudimentary to > 0.2 mm wide along the lateral edges of the sporangium. Sclerified phyllopodia present. Sporangium at the base of the leaf 2.5–3.5 × 2.0–2.5 mm, oblong, black or reddish-black, concolorous. Megaspores 320‒390 µm in diameter (average = 350 µm, *n* = 20), trilete, white, not lustrous; proximal and distal surfaces sparsely verrucate, macrosculpture 10‒25 × 19‒31 µm, slightly wider than high; laesurae 26‒30 × 16‒21 µm, slightly higher than wide. Microspores 23‒27 µm long (average = 25 µm, *n* = 20), light brown, monolete, proximal surface smooth, distal surface sparsely echinate.

#### Distribution and Habitat.

*Isoetes santacruzensis* is only known from its type locality, where it grows as aquatic to ephemeral terrestrial in rocky granite outcrops, at elevations of about 450 m.

#### Etymology.

The specific epithet refers to the type region, the Department of Santa Cruz in Bolivia (Fig. 3).

#### Notes.

**Figure 4.**
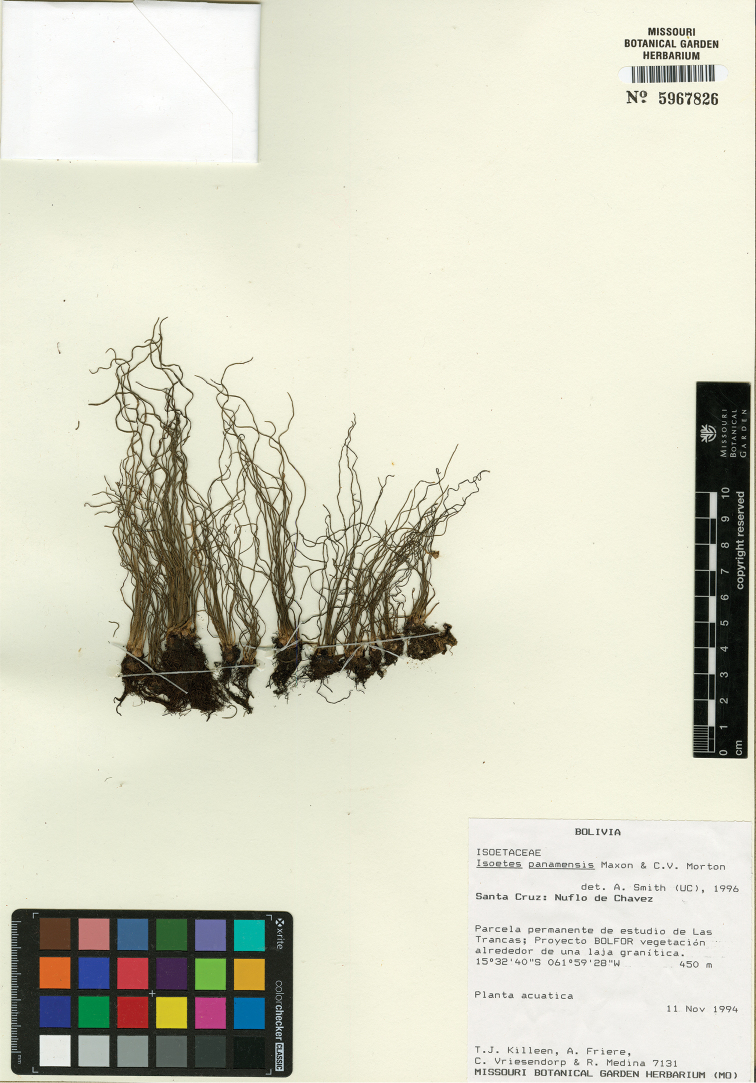
Type of *Isoetes santacruzensis* (image courtesy of the herbarium MO). Photographer: Mike Blomberg.

**Figure 5.**
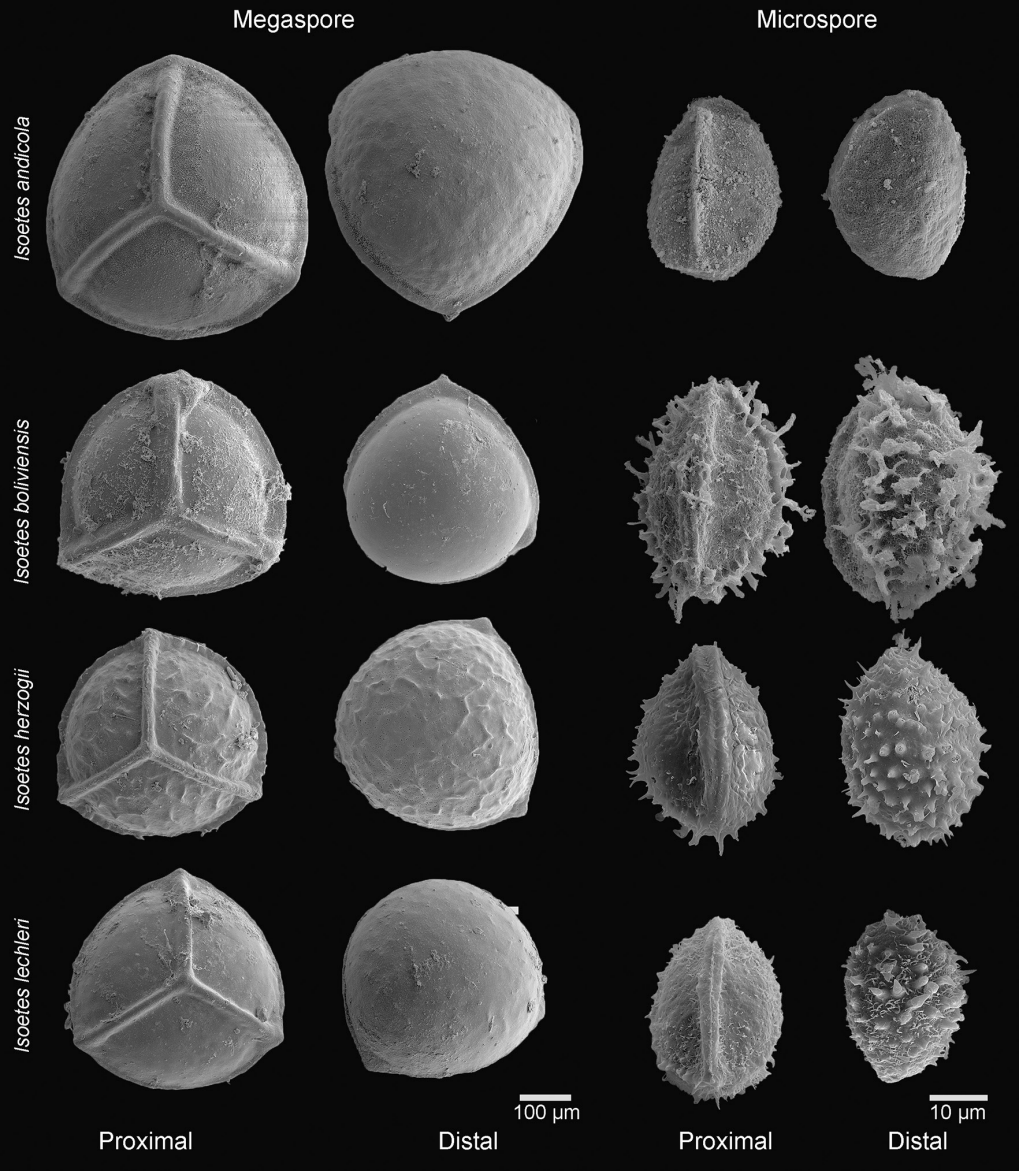
SEM images of the mega- and microspores of *Isoetes andicola* (*Fuentes 13907*, MO), *I. boliviensis* (*Casas 6619*, MO), *I. herzogii* (*Ritter 2209*, MO) and *I. lechleri* (*Solomon 15517*, MO).

**Figure 6.**
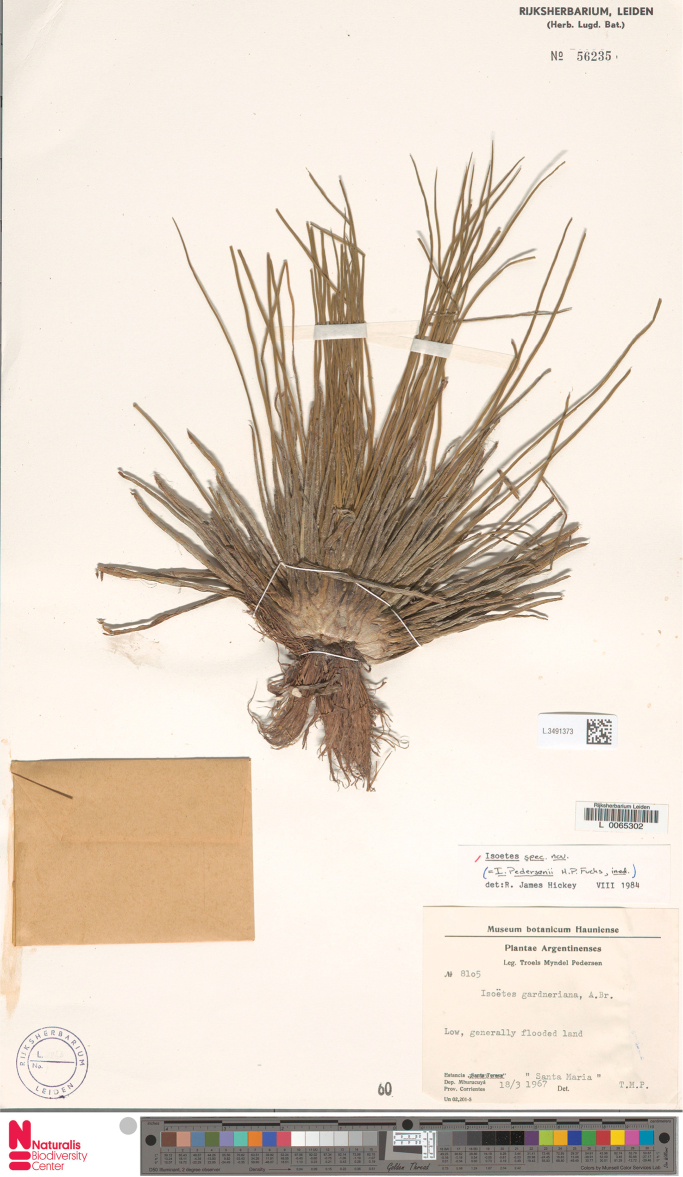
Type of *Isoetes pedersenii* (image courtesy of the herbarium L). Photographer: Christel Schollaardt.

Until now, six species of *Isoetes* were known from Bolivia, although the presence of unpublished species has already been mentioned (Kessler and Smith 2018). Most of the known Bolivian species are from Andean habitats (Kessler and Smith 2018) and have rugulate, laevigate (Fig. 5) or tuberculate megaspores (see Hickey 1986b, Fig. 2) and laevigate, echinate or tuberculate microspores (Fig. 5). Besides habitat, the macrosculpture of at least one of the spore types, megaspore or microspore, helps to differentiate *I. santacruzensis* from the Andean *Isoetes* species (Figs 2, 4). Additionally, *I. santacruzensis* is similar to *I. pedersenii* by its small and verrucate megaspores. However, *I. santacruzensis* can be readily distinguished by its erect and flexuous leaves (vs. ascending, linear and straight; Fig. 6), as well as by the characters present in the taxonomic key.

#### Conservation status.

*Isoetes santacruzensis* is currently known from a single locality. The expansion of agricultural activities and cattle farming in this area show that this species may be prone to the effects of human activities within a very short time. However, given its potential occurrence in other areas and the lack of current knowledge about its distribution range, *I. santacruzensis* should be assessed as data deficient (DD), according to IUCN criteria (IUCN 2012).

### Key to the species from the Brazilian Pantanal wetlands and Bolivia

**Table:** 

1	Plants from lowlands < 500 m	**2**
–	Plants from Andean highlands 2500–5200 m	**5**
2	Megaspores baculate (more rarely tuberculate)	***Isoetes panamensiss.l***.
–	Megaspores pustulate or verrucate	**3**
3	Plant aquatic submerged; megaspores pustulate; laesurae of the megaspore at least four times wider than high	***Isoetes dubsii***
–	Plant amphibious or terrestrial; megaspores verrucate; laesurae of the megaspore slightly wider than high	**4**
4	Leaves ascending, straight; sclerified phyllopodia absent; sporangium hyaline; megaspore densely verrucate on the distal surface; microspore densely echinate	***Isoetes pedersenii***
–	Leaves erect, flexuous; sclerified phyllopodia present; sporangium black or reddish-black; megaspores sparsely verrucate on the distal surface; microspores sparsely echinate	***Isoetes santacruzensis***
5	Plants of cushion bogs; stem vertically elongate; leaves 50–200 per individual	***I. andicola***
–	Plants of lakes, pools, streams and marshes; stem globose; leaves < 50 per individual	**6**
6	Foliar gemmae present; sclerifed phyllopodia present	***I. eshbaughii***
–	Foliar gemmae absent; sclerifed phyllopodia absent	**7**
7	Leaves flaccid, lax to weakly erect; microspores verrucate or tuberculate on the distal surface	***I. boliviensis***
–	Leaves turgid, stiffly erect; microspores echinate on the distal surface	**8**
8	Leaves without dark pigmentation basally; megaspores rugulate (rarely smooth)	***I. herzogii***
–	Leaves usually with dark brown to nearly sclerotic pigmentation basally; megaspores smooth	***I. lechleri***

## Supplementary Material

XML Treatment for Isoetes dubsii

XML Treatment for Isoetes santacruzensis
